# Interfacial Electromigration for Analysis of Biofluid Lipids in Small Volumes

**DOI:** 10.1021/acs.analchem.3c04309

**Published:** 2023-12-05

**Authors:** Madison E. Edwards, Dallas P. Freitas, Erin A. Hirtzel, Nicholas White, Hongying Wang, Laurie A. Davidson, Robert S. Chapkin, Yuxiang Sun, Xin Yan

**Affiliations:** † Department of Chemistry, Texas A&M University, 580 Ross Street, College Station, Texas 77843, United States; ‡ Department of Nutrition, Texas A&M University, 373 Olsen Blvd, College Station, Texas 77845, United States

## Abstract

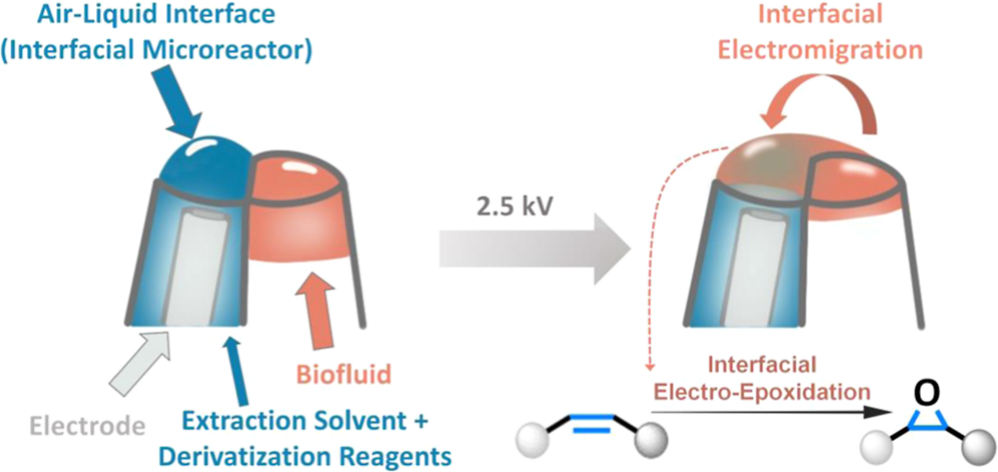

Lipids are important biomarkers within the field of disease diagnostics and can serve as indicators of disease progression and predictors of treatment effectiveness. Although lipids can provide important insight into how diseases initiate and progress, mass spectrometric methods for lipid characterization and profiling are limited due to lipid structural diversity, particularly the presence of various lipid isomers. Moreover, the difficulty of handling small-volume samples exacerbates the intricacies of biological analyses. In this work, we have developed a strategy that electromigrates a thin film of a small-volume biological sample directly to the air–liquid interface formed at the tip of a theta capillary. Importantly, we seamlessly integrated in situ biological lipid extraction with accelerated chemical derivatization, enabled by the air–liquid interface, and conducted isomeric structural characterization within a unified platform utilizing theta capillary nanoelectrospray ionization mass spectrometry, all tailored for small-volume sample analysis. We applied this unified platform to the analysis of lipids from small-volume human plasma and Alzheimer’s disease mouse serum samples. Accelerated electro-epoxidation of unsaturated lipids at the interface allowed us to characterize lipid double-bond positional isomers. The unique application of electromigration of a thin film to the air–liquid interface in combination with accelerated interfacial reactions holds great potential in small-volume sample analysis for disease diagnosis and prevention.

## Introduction

Lipids are biomolecules that serve as the building blocks for all living cells and are essential to cellular functions of energy storage and cell signaling.^1,2^ Dysregulated lipid metabolism has been observed to contribute to the development and progression of many diseases, including cancers,^1,3,4^ cardiovascular disease,^5−7^ and Alzheimer’s disease.^8−12^ Biological fluids such as blood (including serum and plasma), urine, saliva, tears, and cerebrospinal fluid offer ease of collection with minimal or no discomfort to the patient.^13^ Lipid changes have been detected in biological fluids, which provide a ready footprint of the metabolic alterations in diseases, enabling them to be used as markers for diagnosing disease, monitoring disease progression, and evaluating treatment effectiveness.^3,11,14−18^ Small-volume detection is desirable for biofluid analysis as it offers remarkable advantages such as portability, inexpensiveness, capacity for mass production, and unique applicability in the analysis of fluids with limited volumes, including those from the eye, blisters, and the cerebrospinal area.^13,19^

Mass spectrometry (MS) is valuable for lipid analysis due to its identification and quantitation capabilities in analyzing complex samples.^20,21^ Tandem MS (MS/MS) via collision-induced dissociation (CID) can provide structural information on lipid head groups and fatty acyl chains^22,23^ but has limited capability in determining carbon–carbon double bond (C=C) positional isomers because low-energy CID cannot produce fragment ions to locate C=C bonds.^24^ Importantly, recent efforts have enabled lipid characterization at the isomer level, including the development of novel ion activation methods,^25−31^ chemical derivatization methods,^32−38^ and coupling with ion mobility spectrometry.^39−43^ Our group developed an interfacial microreactor to accelerate electrochemical reactions and enable in situ lipid analysis with C=C resolution.^44,45^ The interfacial microreactor was incorporated in a single-barrel electrospray emitter with a large orifice (75–139 μm) formed at the electrified meniscus upon the application of a voltage to the solution (2 kV) lower than the electrospray voltage (3 kV). The meniscus provided a large air–liquid interface for interfacial acceleration^46,47^ while maintaining contact between lipids and the electrode, allowing acceleration of electro-epoxidation of unsaturated lipids, which produced diagnostic fragments to assign C=C positions.^44,45,48,49^ Prior to analysis using the above methods, lipid extraction from complex samples, including biofluids, is usually required using methods, such as Folch et al.,^50^ Bligh & Dyer,^51^ and Matyash et al.,^52^ which are step-heavy, requiring large volume samples, and timely processes and therefore are not compatible with small-volume biofluid sample analysis.

**Figure 1:**
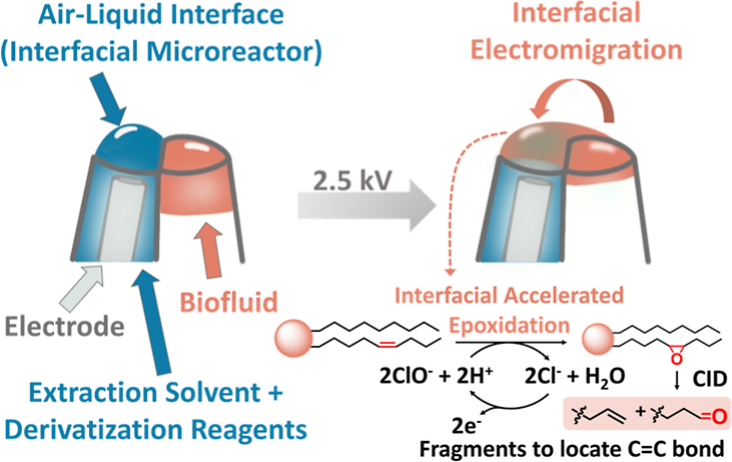
Interfacial electromigration of a small-volume biofluid sample from the barrel without an electrode to the barrel with an electrode in a theta capillary, where accelerated interfacial electro-epoxidation of extracted unsaturated lipids occurs. Fragmentation of the epoxidation product upon CID generates fragment ions, allowing the determination of the lipid C=C bond position.

In this work, we developed a strategy that combines in situ extraction, accelerated derivatization, and isomeric structure characterization of small-volume biofluid lipids in a single step. This is achieved by electromigrating a thin layer of a small-volume biofluid sample to the interface formed in one of the barrels in a large orifice theta capillary (80 μm), followed by lipid extraction and accelerated electrochemical lipid derivatization occurring at the air–liquid interface (Figure 1). The extracted and derivatized lipids are sprayed into the mass spectrometer and fragmented by CID for structural characterization at the isomer level.

Theta capillaries have two barrels separated by a septum, keeping two solutions from mixing until they are sprayed by electrospray ionization (ESI).^53−56^ Interestingly, we found that a thin film of liquid could be electromigrated at the interface from one barrel to the other by applying a voltage just below the voltage necessary to initiate ESI. It is worth noting that electromigration of a thin film is unique to the large-orifice theta capillaries and not observed in traditional small ones (10 μm or below). We take advantage of the thin-film migration for in situ extraction of small-volume biological samples and have accelerated lipid reactions at the interface for lipid isomer characterization at extremely low quantities. Interfacial electromigration allows small-volume samples delivered to the interface for dramatic reaction acceleration and enables low-volume (<0.1 μL, e.g., 0.034 mg) biological samples to be derivatized for in-depth structural analysis.

## Experimental Section

### Materials and Reagents

All reagents were of analytical or chromatographic grade and used without further purification. Acetonitrile (ACN), water (H_2_O), chloroform (CHCl_3_), methanol (MeOH), hydrochloric acid (HCl), ammonium chloride (NH_4_Cl), ethyl acetate (EtOAc), and thioflavin S. Methyl *tert*-butyl ether (MTBE), and live cell imaging solution were purchased from Thermo Fisher Scientific (Waltham, MA). All lipid standards were purchased from Avanti Polar Lipids, Inc. (Alabaster, AL). Pooled normal human plasma with anticoagulant lithium heparin was purchased from Innovative Research, Inc. (MI, USA) with a certificate of analysis. All reagents and lipids were used without any additional purification.

### Animals

Breeders of 5× FAD mice were purchased from Jackson Laboratory (MRRC strain no. 034,848-JAX; Stock# 008730). Heterozygous 5× FAD mice were used for experiments. Food and water were provided ad libitum. All experimental procedures were approved by the Institutional Animal Care and Use Committee at Texas A&M University (IACUC 2022-0180), and all methods were performed in accordance with the relevant guidelines and regulations. To collect mouse brains, mice were first anesthetized using isoflurane and were then humanely euthanized for organ sample collection. Whole blood was collected from 9 months old 5× FAD mice and their GHS-R knockout counterparts. The blood sat at room temperature for 30 min, serum was isolated by centrifugation at 3000 rpm for 5 min, then stored at −80 °C for analysis.

### Nomenclature

This paper follows the lipid nomenclature guidelines from Liebisch et al.^57^ C=C bond position is indicated using the Δ-nomenclature system. Additionally, *sn*-isomers are indicated with a “/” if sn position is known, or “_” if sn position is unknown. For example, PC 16:0_18:1 (Δ6) indicated that the sn position is not determined, and the unsaturation (C=C bond) lies between carbons 6 and 7.

### Fabrication of Theta Tip Capillary

World Precision Instrument (Sarasota, FL) Septum Theta capillaries were used for theta tip fabrication using a micropipet puller (P-1000, Sutter Instruments, Novato, Ca). The following parameters were used to pull a theta tip capillary with an orifice size of 80 μm: heat = 550, pull = 0, velocity = 5, time = 250, pressure = 250, and ramp = 520.

### Mass Spectrometry

MS data were acquired on an Orbitrap Velos Pro mass spectrometer (Thermo Fisher Scientific, San Jose, CA) and an LTQ XL linear ion trap mass spectrometer (Thermo Fisher Scientific, San Jose, CA). All data were analyzed by using the Qual Browser feature of the Xcalibur program (Thermo Fisher Scientific, San Jose, CA). Samples were ionized through applications of 1.5–5 kV AC or DC voltages. An S-lens RF level of 67.9% was used for both ion modes with a capillary temperature of 280 °C. Full MS scans were acquired at *m*/*z* ranges of 50–1000 with a resolving resolution of 60,000, 2 microscans, and a maximum injection time of 200 ms. Tandem mass spectra were obtained via CID with collision energy ranging from 15 to 35 arbitrary units for all data.

### Fluorescence Microscopy

A Nikon Eclipse TS100 instrument (Tokyo, Japan) was used with a mercury excitation lamp for fluorescence excitation. A Nikon UV-2A excitation filter was used to excite thioflavin S at wavelengths of 330–380 nm. The medium-width bandpass filter was coupled to a long-pass barrier filter (cut-on wavelength 420 nm) to allow for a broad range of collection. The microscope was equipped with a Nikon D7200 digital camera to obtain the fluorescence images. A high-voltage power supply from Burle (type PF1055, Lancaster, PA) was used to supply a direct current voltage to the electrode.

### Loading Theta Capillary

A homemade loading source was assembled using a PEEK union (Idex, sold by Thermo Fisher Scientific, Waltham, MA), 0.007″ ID × 1/16″ outer diameter (OD) Nanotight tubing (Cole-Parmer, Vernon Hills, IL), 0.015″ inner diameter (ID) × 1/16″ OD Nanotight tubing (Idex, sold by Thermo Fisher Scientific, Waltham, MA), and 50 μm ID × 150 μm OD fused silica tubing (BGB, Alexandria, VA). Different sizes of capillaries were tested, and the 50 μm ID × 150 μm OD fused silica was chosen, as the solution would not transfer between barrels when loading the theta tip.

### Formation of an Air–Liquid Interface (Interfacial Microreactor) in a Large-Orifice Theta Capillary

**Figure 2:**
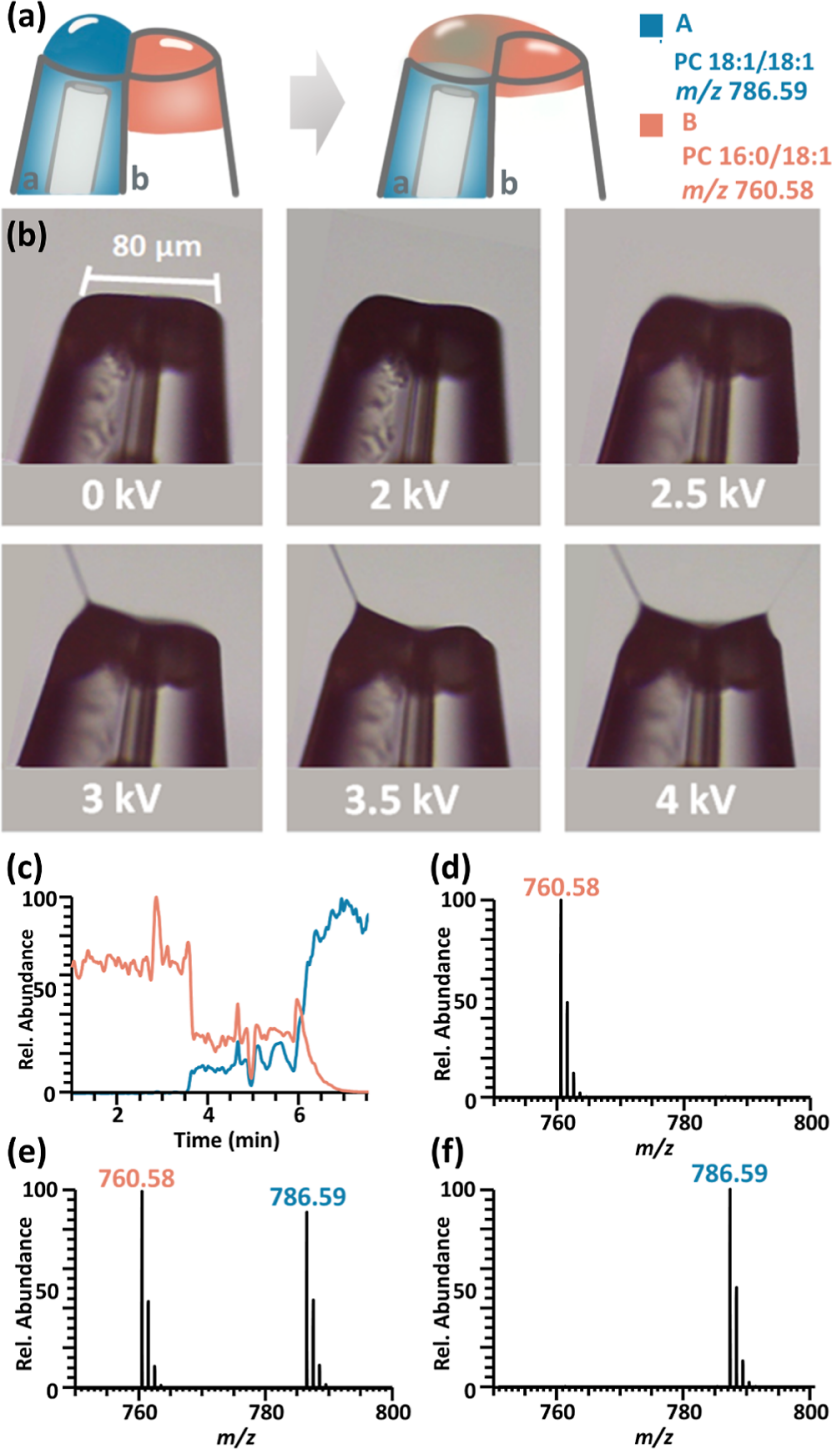
(a) Overview of electromigration in a theta capillary. (b) Spraying modes were achieved at various voltages with the electrode in the left barrel. (c) Selected ion chromatograms of standard **A** PC 18:1_18:1 (10 μL, 50 μM with 0.01% formic acid) loaded into barrel **a** and shown at *m*/*z* 786.59 and standard **B** PC 16:0_18:1 (1 μL, 50 μM with 0.01% formic acid) loaded into barrel **b** and shown at *m*/*z* 760.58. Mass spectra were collected at times after (d) 1, (e) 5, and (f) 8 min.

A mixture of ACN/H_2_O (v/v = 1:1) was loaded into both barrels of a large-orifice theta tip. A high-voltage power supply from Burle (type PF1055, Lancaster, PA) was used to supply a direct current voltage to a platinum (Pt) wire electrode in one barrel of the theta emitter. Images depicting the formation of the theta-tip interfacial microreactor (Figure 2b) were taken on an OMAX microscope (China).

### Electromigration of a Thin Film

Lipid standards (PC 18:1_18:1 and PC 16:0_18:1) were prepared in ACN and diluted to a concentration of 100 μM. PC 18:1_18:1 was loaded into one barrel along with a Pt electrode, and PC 16:0_18:1 was loaded into the other barrel. Upon the application of a voltage tuned between 2.8 and 3.2 kV, we were able to observe in the mass spectrometer a high abundance of the PC 16:0_18:1 standard in the barrel without the electrode. This shows the migration of the solution from the barrel without an electrode to the meniscus of the barrel with the electrode and the importance of the electrode placement (Figure S1).

## Results and Discussion

### Delivery of a Liquid Thin Film to the Interface Via Electromigration in a Theta Capillary

We found that a liquid thin film could be delivered to an air–liquid interface by using the setup shown in Figure 2a. In a theta capillary with an orifice of 80 μm, barrel **a** was fitted with a Pt electrode immersed in a standard **A** solution (10 μL of PC 18:1_18:1 at *m*/*z* 786, 50 μM with 0.01% formic acid), and barrel **b** was loaded with a standard **B** solution (1 μL of PC 16:0_18:1 at *m*/*z* 760, 50 μM with 0.01% formic acid). The two molecules **A** and **B** do not react and are used to show the flow direction. We first formed a large air–liquid interface in barrel **a** by applying 2 kV to solution **A** (Figure 2b recorded with a microscope camera). When we increased the voltage to 2.5 kV, surprisingly, a thin film was observed moving from barrel **b** to **a**. The flow direction was determined by monitoring **A** and **B** using MS. Interestingly, **B** was first shown in the mass spectrum through the Taylor cone formed at barrel **a** (Figure 2c–f) followed by the appearance of **A**, indicating that the liquid flow was from **b** to **a** and the liquid transferred was restricted to the interface. Switching the standard solutions in the two barrels led to the same conclusion (Figure S1). Under the electromigration condition, the application of a high voltage (+2.5 kV, as shown in Figure 2b) to the electrode causes the formation of a rounded liquid meniscus in barrel **a** with the electrode. This occurs prior to its deformation into a Taylor cone driven by charge accumulation at the air–liquid interface. In contrast, barrel **b**, lacking an electrode, experiences a much weaker induced electric field. This results in the formation of a thin layer that joins with the meniscus in barrel **a**. Ions can then migrate from barrel **b** to barrel **a** through the thin film, establishing a connection with the meniscus. The flow rate of theta capillary spray was determined to be 103.17 ± 22.45 nL/min (Table S1). Utilizing the migration time of 0.045 min (Figure S2), we calculated the volume of the thin film migrated as 4.64 ± 1.0 nL (Table S2).

The electromigration of a thin film to the interface only occurred in large orifice theta glass capillaries using relatively high voltages (40 μm diameter for each barrel, optimally 2.5–3.2 kV) and is different from electroosmosis^54^ occurring in small orifice capillaries using low voltages (5–10 μm diameter, 300–500 V), which transfer the solution from barrel **a** to **b**. We observed the opposite liquid flow in these two types of capillaries using the zwitterion dye thioflavin S solution (Figure S3). In addition, two Taylor cones were observed in the large orifice theta capillary *n*_ESI_ when higher voltages (4 kV) were applied (Figure 2b).

### Electromigration for In Situ Lipid Extraction from Human Plasma

We subsequently assessed the application of electromigration to achieve in situ extraction and profiling of lipids from small-volume human plasma (<0.1 μL, ∼0.034 mg, pooled normal human plasma from Innovative Research, Inc.), thus avoiding the use of traditional lipid extraction. Human plasma was chosen for analysis as it is known to contain lipids that are biomarkers for disease diagnostics and are readily available.^12,58,59^

**Figure 3:**
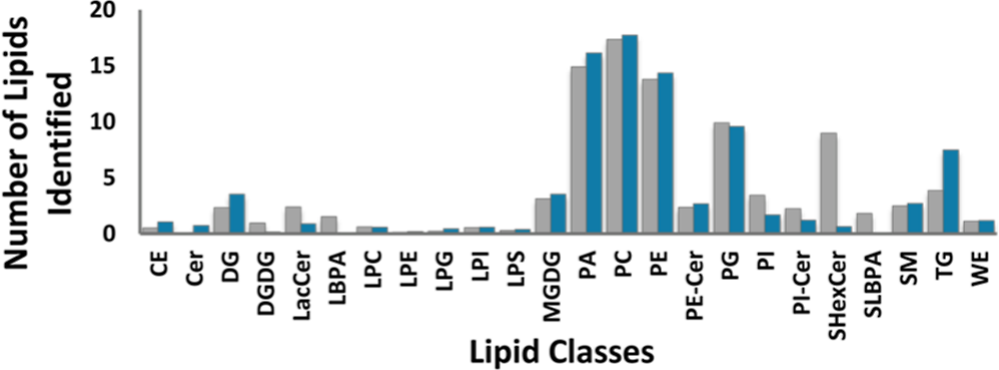
Small-volume human plasma lipid profiling via electromigration and in situ extraction in the theta capillary coupled with MS analysis. A lipid profile comparison was generated between the use of Matyash solvents (gray bar) and the modified Matyash solvents (blue bar).

In a theta capillary, we loaded human plasma (<0.1 μL, ∼0.034 mg) into barrel **b** and the spray solvent into barrel **a**. After a voltage of 2.5 kV was applied, the meniscus of the air–liquid interface formed. Subsequently, a thin film of minimal human plasma was electromigrated from its barrel directly onto the meniscus at 3 kV. This allowed a small amount of mixing between the sample and solvent to occur only at the interface where the lipid extraction took place. Following the in situ extraction, lipids were then detected via MS as a very fine plume of charged droplets was released from the meniscus. The lipid profile and identified lipids from the human plasma are shown in Figures 3 and S4. Lipids of 25 classes have been identified, including polar and nonpolar lipids (Tables S3 and S4). Glycerophospholipids such as phosphatidylcholine (PC), phosphatidic acid (PA), phosphatidylethanolamine (PE), phosphatidylinositol (PI), phosphatidylglycerol (PG), and triglycerides (TG) dominated the profile. A modified Matyash solvent, which contained MTBE/ACN/H_2_O (v/v = 40:4:1) with 10 mM of NH_4_Cl and 1 mM HCl was used as the optimal extraction/spray solvent after comparing it to traditional Matyash solvent (Figure 3) and other systems (Table S5). The addition of NH_4_Cl was helpful in identifying nonpolar lipids such as cholesteryl esters (CE), diacylglycerol (DG), and TG. Electromigration allowed the lipid to be observed in MS with a limit of detection of 10 fM (Figure S5).

### Electromigration Combined with the Electrochemical Interfacial Microreactor for In Situ Extraction and Characterization of Fatty Acids from Mouse Serum at the Isomer Level

Lipid derivatization is an appealing strategy for isomer identification when coupled with tandem MS as it involves minimal instrument modification, and reagents are often easy to access. Our previous work shows that voltage-controlled electro-epoxidation of lipid C=C bonds can be achieved in the single-barrel interfacial microreactor to characterize the C=C bond positions in unsaturated lipids.^44,60^ After loading lipids in ACN and H_2_O in the presence of HCl into the interfacial microreactor, the chloride in an acidic environment was oxidized to hypochlorite, which is used to aid the epoxidation of the lipid double bond into an epoxide.^44,45^ Herein, we combine electromigration and an electrochemical interfacial microreactor to derivatize lipids extracted in situ from small-volume mouse serum samples for lipid characterization at the isomer level.

**Figure 4:**
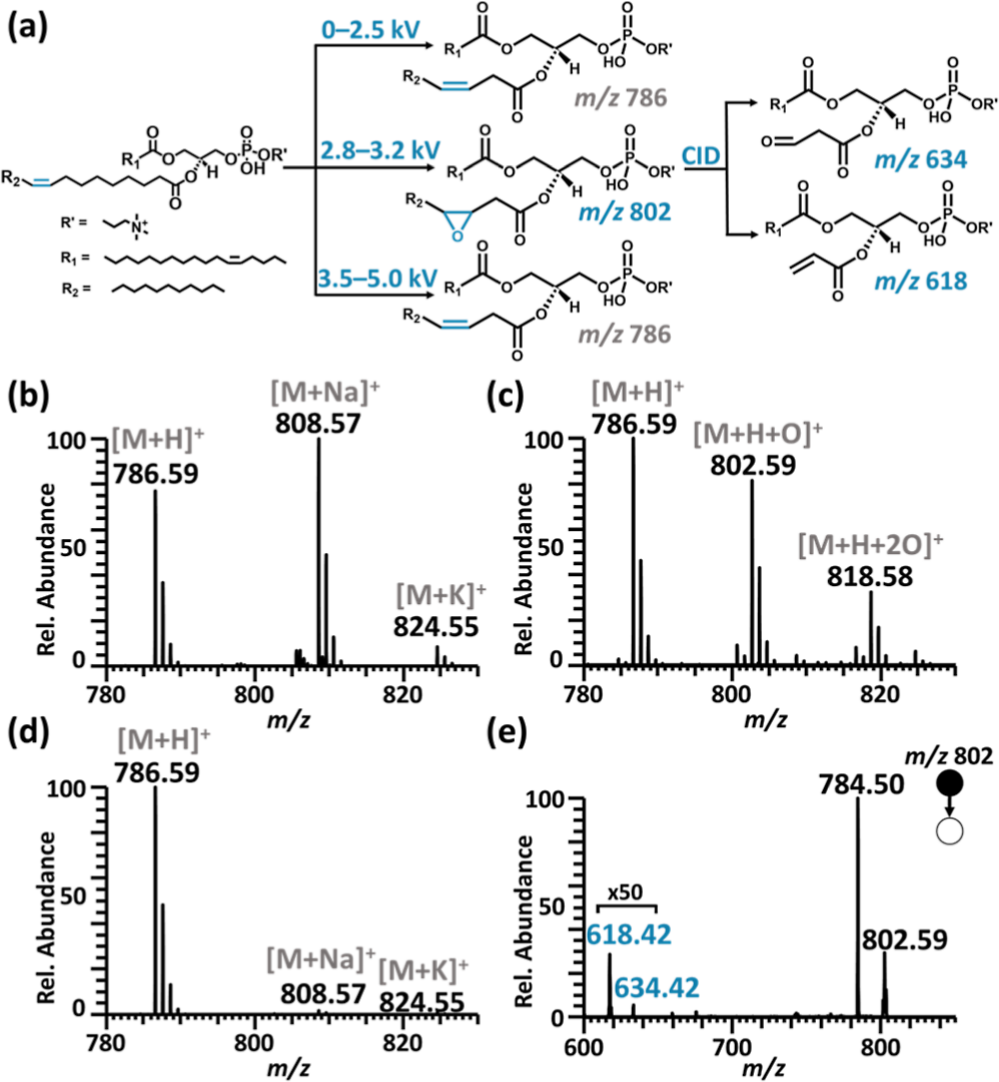
Interfacial voltage-controlled electroepoxidation of lipid PC 18:1_18:1 coupled with tandem MS was used to generate diagnostic fragments at *m*/*z* 618 and 634 for lipid C=C bond position determination. (a) Epoxidation scheme showing voltage-dependent product formation and the diagnostic fragment ions upon CID. Mass spectra obtained when voltages of (b) 2.5–2.7; (c) 2.8–3.2; and (d) 3.5 kV were applied to the electrode in the theta capillary containing a solution of lipid standard PC 18:1_18:1. (e) Tandem mass spectrum of electroepoxidation product ions at *m*/*z* 802 upon CID. The diagnostic ions highlighted in blue indicate the C=C bond in PC 18:1_18:1 at the Δ6 position.

We examined the combination of electromigration and electrochemical interfacial reaction using a lipid standard PC 18:1_18:1 (50 μM), which was loaded into one barrel of the theta capillary, and the solvent system (ACN/water, v/v = 4:1, and 10 mM HCl) was loaded into the other barrel with the Pt electrode (Figure 4a–e). When a relatively low voltage was applied (2.5–2.7 kV), PC 18:1_18:1 at *m*/*z* 786 along with its corresponding sodium and potassium salt adducts at *m*/*z* 808 and *m*/*z* 824 were detected (Figure 4a,b). After applying a voltage between 2.8 and 3.2 kV, the interfacial microreactor was formed, and electromigration of the lipid standard occurred. The formation of the monoepoxide at *m*/*z* 802 and the diepoxide at *m*/*z* 818 was observed (Figure 4a,c). Further, when we applied a relatively high voltage (3.5 kV or greater), the interfacial microreactor was no longer formed, and the only species detected was the protonated lipid (Figure 4c). This can be explained by the surface tension of the meniscus (interfacial microreactor) being overcome by Coulombic forces and the rounded meniscus becoming inverted into a jet of liquid, which diminished the interfacial microreactor.^13^ The formation of the epoxide at the lipid C=C bond allows CID to differentiate diagnostic ions for characterization. The diagnostic ions at *m*/*z* 634 and *m*/*z* 618 indicate that the C=C bond was at the Δ6 position in PC 18:1_18:1 (Figure 4e). After demonstrating the feasibility of coupling electromigration and the interfacial microreactor in the theta capillary, we analyzed lipids from small-volume mouse serum at the C=C position isomer level.

Alzheimer’s disease (AD) is a neurodegenerative disorder with no cure and is progressive, unremitting, and often fatal.^8^ It is characterized by progressive and irreversible damage to brain cells, leading to a decline in cognitive function and memory loss. Recent research has highlighted the potential role of ghrelin, a survival and hunger hormone, produced by the stomach, and its receptor, growth hormone secretagogue receptor (GHS-R), in the pathogenesis of AD.^61^ Studies have suggested that ghrelin and GHS-R may affect lipid metabolism^62,63^ and contribute to the development and progression of AD.^61,64^ To further investigate this potential mechanism, we conducted a study using serum samples from GHS-R knockout 5× FAD (familial Alzheimer’s disease) mice and compared them to normal 5× FAD mice.

**Figure 5:**
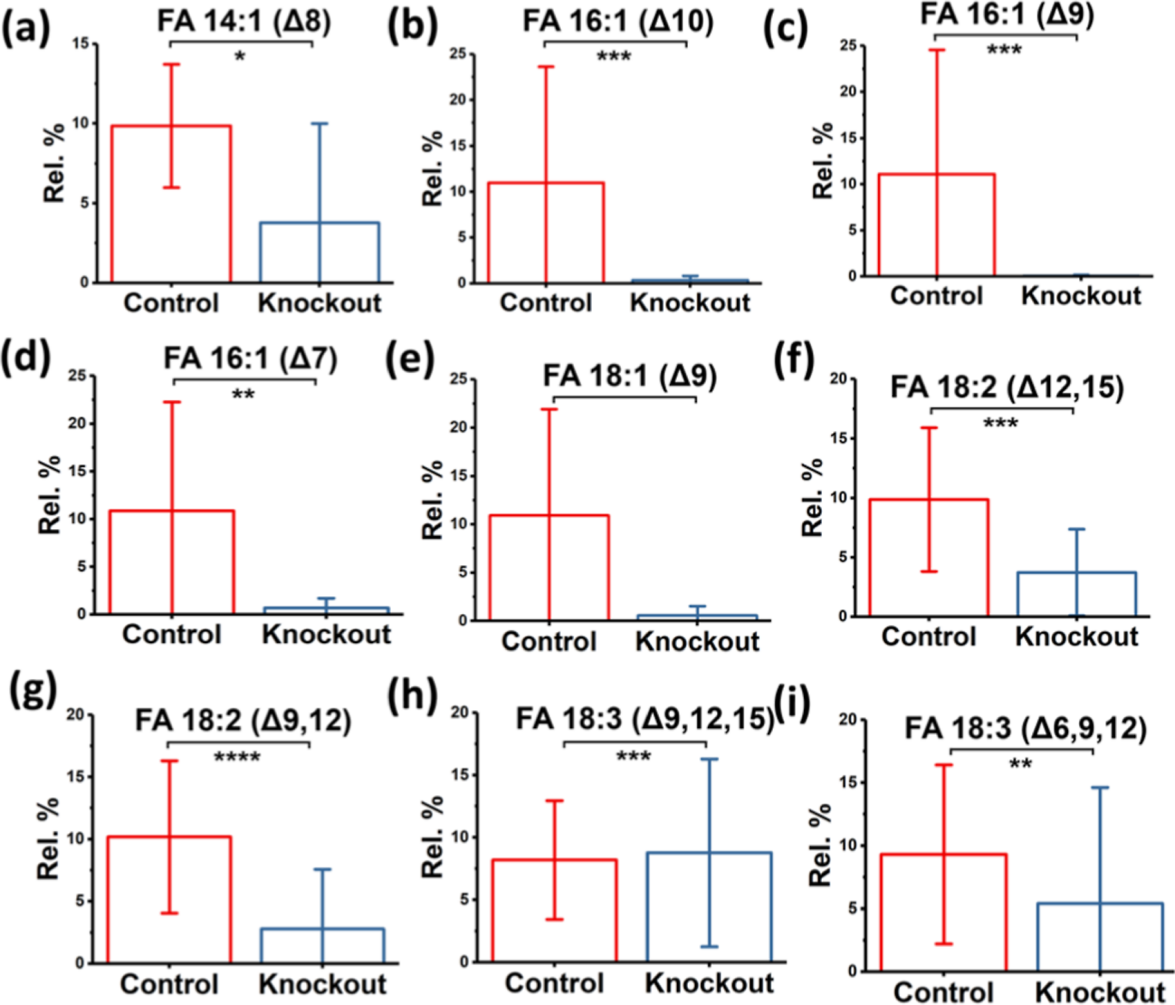
C=C bond positional isomer distribution of (a) FA 14:1 (Δ8), (b) FA 16:1 (Δ10), (c) FA 16:1 (Δ9), (d) FA 16:1 (Δ7), (e) FA 18:1 (Δ9), (f) FA 18:2 (Δ12,15), (g) FA 18:2 (Δ9,12), (h) FA 18:3 (Δ9,12,15), and (i) FA 18:3 (Δ6,9,12) between the GHS-R knockout 5× FAD versus normal 5× FAD mice (control). The distribution is shown in relative percent (%) of isomer intensity. C=C bond positions were determined by lipid electro-epoxidation in the theta interfacial microreactor. Differences between the two groups of samples were evaluated for statistical significance using the student’s *t*-test (*P < 0.2, **P < 0.1, ***P < 0.05, and ****P < 0.01).

To form electro-epoxidation products of serum fatty acids, serum was loaded in one barrel, and alternating current (AC) voltage was applied to the solution of EtOAc, with NH_4_Cl and HCl^34^ loaded in the other barrel containing the electrode. In this way, hypochlorite was formed by anodic oxidation of chloride to enable the epoxidation of FAs in the positive half cycle of the AC voltage after electromigration. This solvent system was chosen for a free (unesterified) FA extraction of the serum since the addition of EtoAC allows for an increased solubility of the FAs in solution.^34^ The unesterified FAs and their electro-epoxidation products were observed in the negative half cycle of the AC voltage. The electro-epoxidation products of unsaturated FAs myristoleic acid (FA 14:1), palmitoleic acid (FA 16:1), alpha/gamma linolenic acid (FA 18:3), linoleic acid (FA 18:2), and oleic acid (FA 18:1) were clearly detected in the spectrum via in situ FA extraction and epoxidation (Figure S6). These ions were isolated and fragmented via CID, producing diagnostic ions indicating the C=C bond positions (Figure S7). In total, 23 lipid C=C bond positional isomers were identified (Table S6) after in situ extraction of small-volume mouse serum, followed by electro-epoxidation in the interfacial microreactor and tandem MS analysis. Quantitation of FA C=C bond isomers was achieved by using the intensities of each C=C bond isomer’s diagnostic ions in CID normalized by total ion counts (TIC). This analysis revealed various distributions of C=C bonds between two groups (as illustrated in Figures 5 and S8), with the rare lipids we detected being consistent with previous reports.^65^

Our investigation uncovered notable differences in the abundance of several unesterified fatty acids in GHS-R knockout mice compared to the control group. Specifically, fatty acids such as 14:1 (Δ8), 16:1 (Δ10), 16:1 (Δ9), 16:1 (Δ7), 18:1 (Δ9), 18:2 (Δ12,15), 18:2 (Δ9,12), 18:3 (Δ9,12,15), and 18:3 (Δ6,9,12) exhibited significant variations. Furthermore, we observed distinct distributions of C=C positional isomers within FA 18:2 and FA 18:3, which correspond to isomers of linoleic acid and alpha-linolenic acid, respectively. Our understanding of the role of lipids in AD onset and progression has primarily focused on identifying lipid classes rather than delving into isomeric information.^66^ However, gaining insight into the isomeric structure and function of lipids is crucial for comprehending how their distribution changes in the presence of specific diseases.^67^ Thus, it is noteworthy that our analysis of the serum lipidome in GHS-R knockout 5× FAD mice revealed dysregulation of unesterified fatty acids at the isomer level, underscoring the significance of ghrelin and GHS-R in regulating lipid metabolism in the context of AD.

## Conclusions

In this study, we have developed a strategy of delivering a liquid thin film to an air–liquid interface using voltage-controlled electromigration in a theta capillary. Electromigration was characterized by tracking the liquid flow using lipid standards and dye. The electromigration coupled with an interfacial microreactor promoted reaction acceleration at the interface. We also demonstrate its powerful application in small-volume in situ extraction and derivatization for analyzing lipids at the isomer level using a small quantity of biological samples.

## Supporting Information Available

Electromigration of a thin film in a theta capillary; volume of thin film migrated via electromigration; tracking liquid flow in electromigration using the dye Thioflavin S; in situ extraction of lipids from pooled normal human plasma via electromigration; characterization of fatty acids at the isomer level by electroepoxidation in the interfacial microreactor after electromigration; acceleration of epoxidation of negatively charged fatty acids in mouse serum; and changes of C=C bond positional isomer ratios in the GHS-R knockout 5xFAD mouse serum (PDF)
Measurement of flow rate in the theta capillary spray; volume of thin film migrated; list of identified lipids from human plasma using a modified Matyash extraction method; solvent systems tested for in situ lipid extraction; and lipid isomers that were detected via in situ extraction and epoxidation (XLSX)
