# Supplementary material for: Interfacial Electromigration for Analysis of Biofluid Lipids in Small Volumes
Source: Anal Chem. 2023 Dec 5;95(50):18557–63. doi: 10.1021/acs.analchem.3c04309 (PMC10862378; doi:10.1021/acs.analchem.3c04309)
Supplement: Supplementary file 1 — ac3c04309_si_001.pdf [file ac3c04309_si_001.pdf]

Supporting Information

Interfacial Electromigration for Analysis of Biofluid Lipids in Small Volumes

Madison E. Edwards,[a] Dallas P. Freitas,[a] Erin A. Hirtzel,[a] Nicholas White,[a] Hongying Wang,[b] Laurie A. Davidson,[b] Robert S. Chapkin,[b] Yuxiang Sun,[b] and Xin Yan[a,*]

[a.] *Department of Chemistry, Texas A&M University, 580 Ross St., College Station, TX 77843 (USA)*
[b.] *Department of Nutrition, Texas A&M University, 373 Olsen Blvd., College Station, TX 77845 (USA)*
* *Corresponding author: xyan@tamu.edu*

Table of Content

S1. Electromigration of a thin film in a theta capillary

S2. Volume of thin film migrated via electromigration

S3. Tracking liquid flow in electromigration using the dye Thioflavin S

S4. In situ extraction of lipids from pooled normal human plasma (Innovative Research, Inc.) via electromigration

S5. Characterization of fatty acids at the isomer level by electroepoxidation in the interfacial microreactor after electromigration

S6. Acceleration of epoxidation of negatively charged fatty acids in mouse serum

S7. Changes of C=C bond positional isomer ratios in the GHS-R knockout 5xFAD mouse serum

S1. Electromigration of a thin film in a theta capillary

Lipid standards (PC(18:1/18:1) and PC(16:0/18:1)) were prepared in ACN and diluted to a concentration of 100 µM. PC(18:1/18:1) was loaded into one barrel along with a Pt electrode, and PC(16:0/18:1) was loaded into the other barrel. Upon the application of voltage tuned between 2.8-3.2 kV, we were able to observe in the mass spectrometer a high abundance of the PC(16:0/18:1) standard in the barrel without the electrode. **Figure S1** shows the migration of solution from the barrel without an electrode to the meniscus of the barrel with the electrode and the importance of the electrode placement.

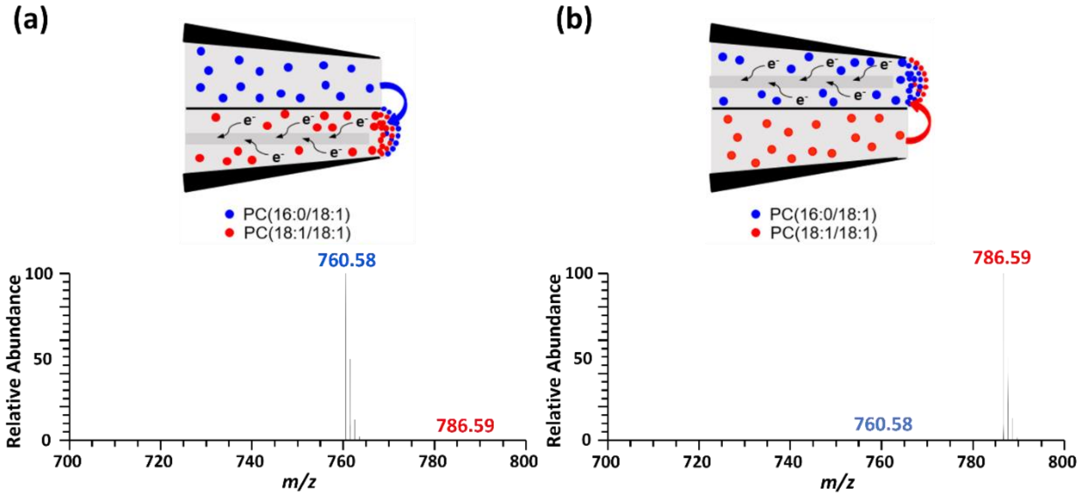

Figure S1. Setup to elucidate the importance of electrode placement on electromigration. (a) The electrode was placed in the barrel with PC(18:1/18:1) and PC(16:0/18:1) was the dominant peak and in (b) the electrode was placed in the barrel with PC(16:0/18:1) and PC(18:1/18:1) was the dominant peak.

S2. Volume of thin film migrated via electromigration

Lipid standards (PC 18:1_18:1 and PC 16:0_18:1) were respectively prepared in ACN and diluted to a concentration of 100 µM. PC 18:1_18:1 was loaded into one barrel with a Pt electrode, and PC 16:0_18:1 was loaded into the other barrel. Upon the application of voltage tuned between 2.8-3.2 kV, we were able to observe the time for a thin film to migrate from the barrel without the electrode to the barrel with the electrode in the extracted chromatogram of PC 16:0_18:1 (**Figure S2**). The volume of thin film migration was calculated to be 4.64±1.0 nL (**Table S2**) based on the spray flow rate which was determined to be 103.17±22.45 nL/min (**Table S1**).

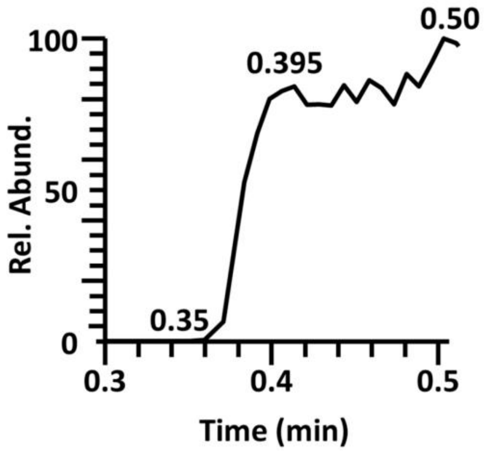

Figure S2. Extracted ion chromatogram for the time measurements used to calculate the volume of thin film migrated via electromigration.

S3. Tracking liquid flow in electromigration using the dye Thioflavin S

Thioflavin S at a concentration of 0.7% diluted in water was loaded into one barrel of a 10 µm theta tip emitter and into one barrel of an 80 µm theta tip emitter (**Figure S3a and c**). Voltage was then applied starting at 0.1 kV and increased in 0.1 kV increments until a voltage of 2.8 kV and 4 kV was achieved for the small and large emitters, respectively. After the application of voltage to the smaller orifice emitter (10 µm), the electroosmosis phenomenon was observed (**Figure S3b**); when voltage was applied to one barrel higher than the other, the solution in the barrel with the higher voltage migrated to the side with the lower voltage (*J. Mass Spectrom.* **2015**, *50* (9), 1063-1070). However, when this same experiment was performed using a large orifice theta tip emitter, a different phenomenon was observed (**Figure S3d**). Using the large orifice theta tip emitter, we observed electromigration where the solution with the lower voltage (in our experiment, the lower voltage barrel received no voltage) migrated to the barrel to which the voltage is applied.

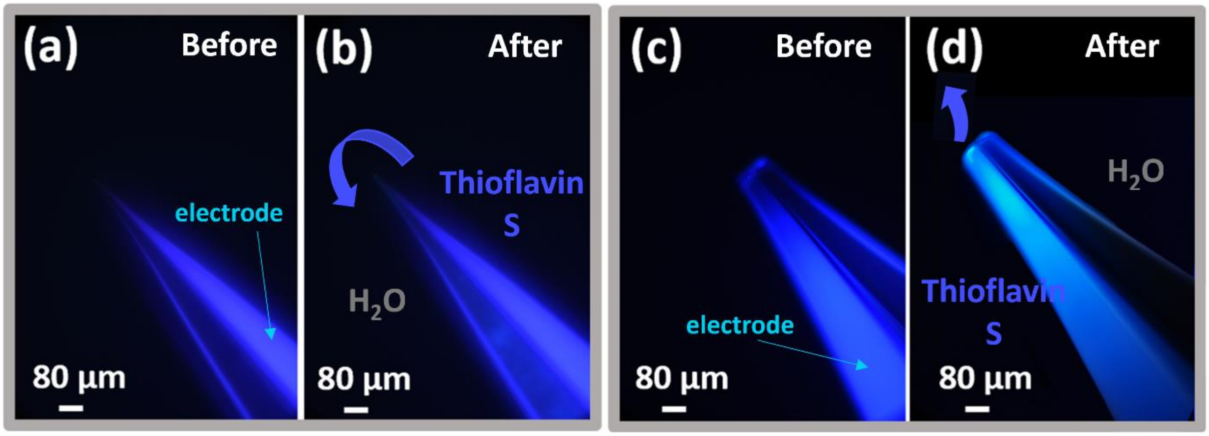

Figure S3. Fluorescence microscopic images showing (a) 10 µm orifice before voltage is applied; (b) electroosmosis, (c) 80 µm orifice before voltage is applied; and (d) electromigration.

S4. In situ extraction of lipids from pooled normal human plasma (Innovative Research, Inc.) via electromigration

Serum (0.1 µL) was pipetted onto a glass microscope slide. The theta capillary was then carefully placed on the droplet of serum and a small amount was placed in the first barrel of the theta emitter via capillarity. The second barrel was then loaded with a modified Matyash solution, which contained MTBE and an ACN/H_2O (4:1, v/v) solution with 10 mM of NH_4Cl, 1mM HCl in a 10:1 volume ratio, and a Pt wire. In-situ lipid extraction was subsequently performed followed by characterization using mass spectrometry.

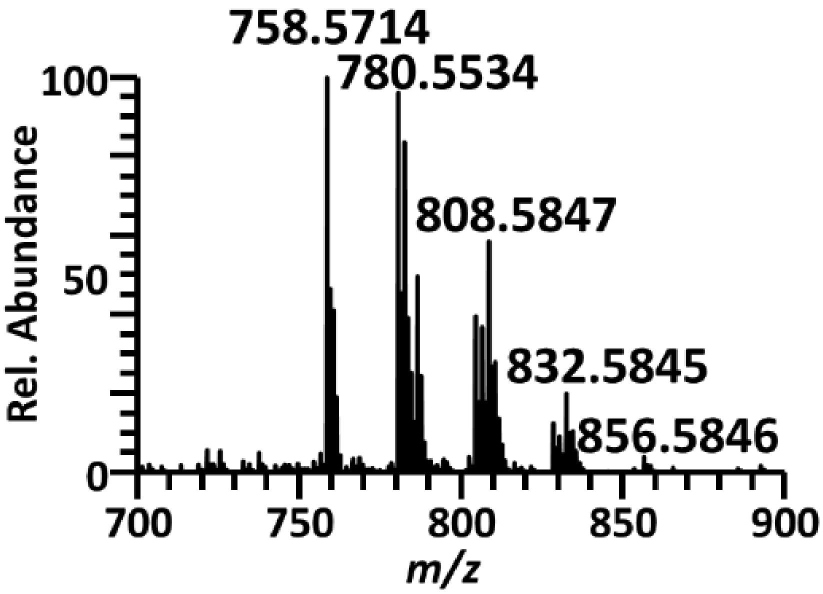

Figure S4. Mass spectrum of human plasma lipids after electromigration and an in situ modified Maytash extraction in a large-orifice theta capillary coupled with mass spectrometry analysis (See Table S3 and S4 for lipid identification).

S5. Characterization of fatty acids at the isomer level by electroepoxidation in the interfacial microreactor after electromigration

S5.1 Determination of detection limit using electromigration in a theta capillary nESI

Lipid standard solutions (PC(18:1/18:1), *m/z* 786 with 0.01% formic acid) at 50 µM, 50 nM, 50 pM, 50 fM, and 10 fM were prepared. Samples were loaded using the same solution into a single barrel nESI emitter (>10 µm orifice size) for comparison to the sample loaded into one barrel of the theta emitter. For the comparison, one barrel of the theta emitter was loaded with the same solution as the single barrel nESI emitter, and the other barrel was loaded with ACN:H_2O (v/v=4:1). Lipid was detected at 10 fM using electromigration with the theta emitter. In comparison, the lipid was not detected using a single barrel nESI emitter at this concentration.

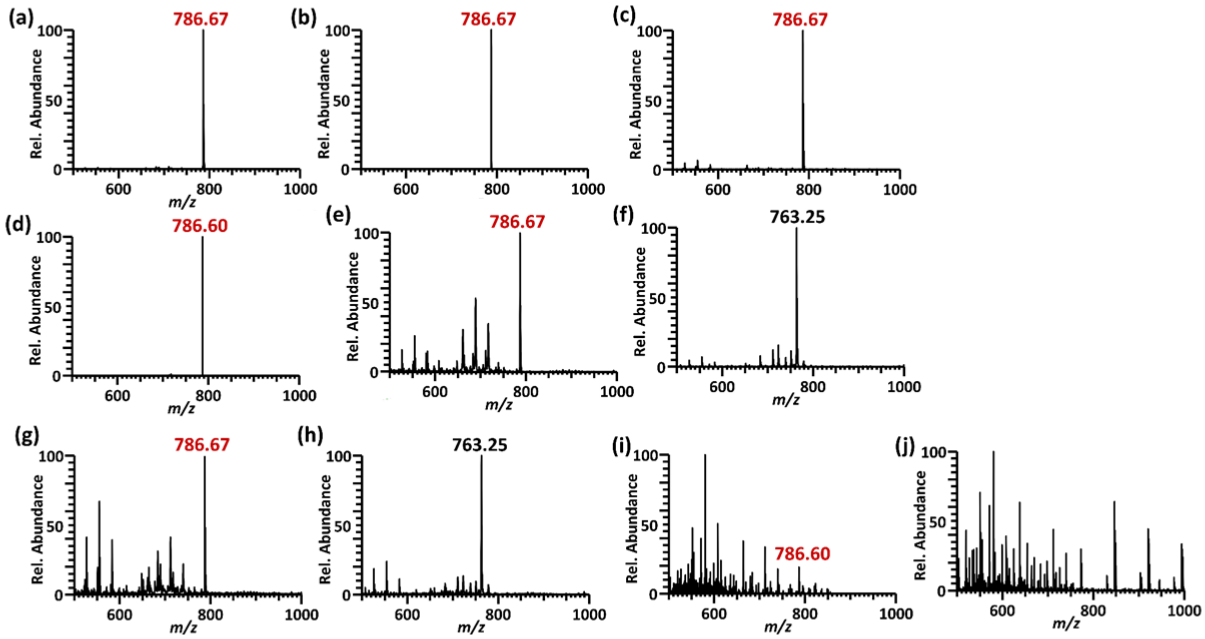

Figure S5. Full mass spectra for theta-emitter (left column) and traditional nESI using an emitter >10 µm (right column). (a) theta emitter, PC(18:1/18:1) at 50 µM, (b) nESI emitter, PC(18:1/18:1) at 50 µM, (c) theta emitter, PC(18:1/18:1) at 50 nM, (d) nESI emitter, PC(18:1/18:1) at 50 nM, (e) theta emitter, PC(18:1/18:1) at 50 pM, (f) nESI emitter, PC(18:1/18:1) at 50 pM, (g) theta emitter, PC(18:1/18:1) at 50 fM, (h) nESI emitter, PC(18:1/18:1) at 50 fM, (i) theta emitter, PC(18:1/18:1) at 10 fM, and (j) nESI emitter, PC(18:1/18:1) at 10 fM.

S5.2 Characterization of fatty acids at the isomer level by electroepoxidation in the interfacial microreactor after electromigration

The theta capillary was placed in front of the MS inlet and a Pt wire was positioned in a barrel containing EtOAC with 10 mM NH_4Cl and 1 mM HCl (Barrel 1). The Pt electrode acts as the working electrode able to provide electrical contact with the spray solvent upon the application of voltage. To initiate electroepoxidation, the voltage was tuned between 2.8-3.2 kV, which was turned off by applying a voltage above 3.5 kV based on the spray modes that occur in the large orifice theta emitter. When the voltage was tuned between 2.8 and 3.2 kV, a meniscus was formed at the tip of the emitter, which is where the epoxidation reaction was accelerated. Serum (0.1 µL) was applied to a microscope slide, and the theta emitter tip was carefully placed on the droplet. Via capillary action, a small amount of the plasma droplet was taken up by the emitter (barrel 2).

S5.3 Investigation of electromigration of lipids with and without mouse serum matrix

We compared the electromigration of lipids with and without mouse serum matrix. In the study of the lipid electromigration in mouse serum, PC 18:1_18:1 and PC 16:0_18:1 were prepared in ACN:H2O (1:1) solvent and in serum, respectively. PC 18:1_18:1 (10 µL) was loaded into barrel **a** with the electrode and PC 16:0_18:1 (0.5 µL) in mouse serum was loaded into barrel **b** without the electrode. Upon the application of voltage to the electrode, we observed a 36 seconds-delay in the electromigration of PC 16:0_18:1 in serum. This could be attributed to the fact that serum is more viscous than the organic solvent which results in the slow migration when applying the same force of migration.

S6. Acceleration of epoxidation of negatively charged fatty acids in mouse serum

An external AC wave function generator (Stanford Research Systems, Sunnyvale, CA) was connected to a digital storage oscilloscope (Hantek Electronic Co., Ltd., Qingdao, China) with a power amplifier (Trek, Lockport, NY). Since the epoxidation reaction was initiated with a positive potential, AC voltage was used to epoxidize the negatively charged fatty acids. A square wave function with the offset=0, frequency=50Hz, and amplitude was increased to apply a tuned voltage of 2.8-3.2 kV to initiate the migration of serum to the meniscus for the in situ extraction and epoxidation.

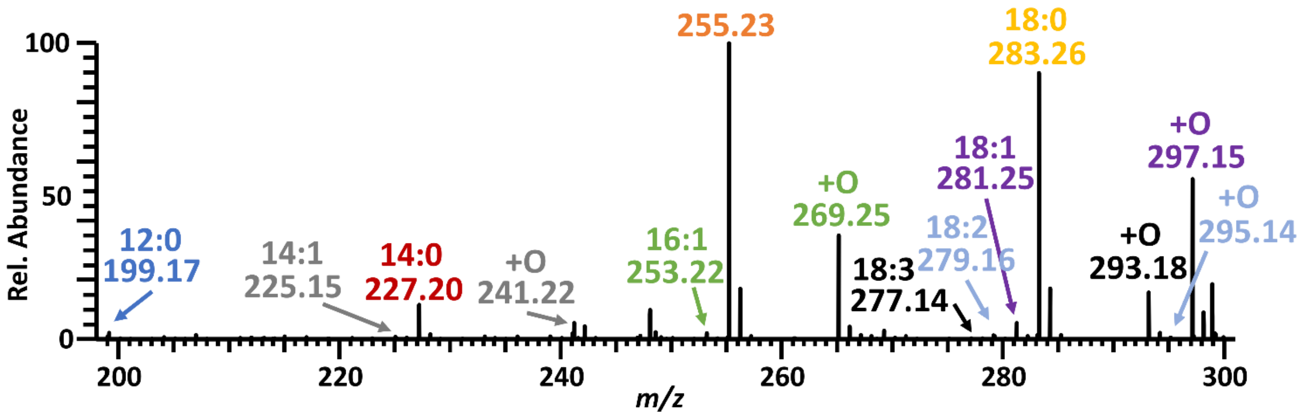

Figure S6. Full mass spectrum showing both protonated and epoxidated FA ions.

The fragmentation of the electro-epoxidation product of the C=C bond positional isomer produces exclusive diagnostic fragment ions for locating the C=C bond while the ion abundance of the fragments is relatively low. This has been observed in previous reports using epoxidation for identifying C=C bond locations. [1, 2] This is in part because of the low fragmentation yield in MS/MS as well as the low ionization efficiency of the diagnostic ions.[2, 3] Our group has been investigating the use of aziridination to increase the ionization efficiency. The addition of nitrogen to the lipid and its corresponding fragments increases abundance of diagnostic ions for C=C bond identification and we extended the methods to both polar and non-polar lipids. However, electroepoxidation was chosen for this experiment, as it can be done electrochemically in the confined volume of the meniscus (see main text), while the aziridination reaction was done in bulk.[4, 5]

References:

1. Feng, Y.; Chen, B.; Yu, Q.; Li, L. Identification of double bond position isomers in unsaturated lipids by m-CPBA epoxidation and mass spectrometry fragmentation. *Anal. Chem.* **2019**, *91* (3), 1791-1795. DOI: 10.1021/acs.analchem.8b04905

2. Tang, S.; Cheng, H.; Yan, X. On-demand electrochemical epoxidation in nano-electrospray ionization mass spectrometry to locate carbon–carbon double bonds. *Angew. Chem. Int. Ed.* **2020**, *59* (1), 209-214. DOI: 10.1002/anie.201911070.

3. Grooms, A. J.; Nordmann, A. N.; Badu-Tawiah, A. K. Plasma-Droplet Reaction Systems: A Direct Mass Spectrometry Approach for Enhanced Characterization of Lipids at Multiple Isomer Levels. *ACS Measurement Science Au* **2022**, *3* (1), 32-44. DOI: 10.1021/acsmeasuresciau.2c00051.

4. Yang, T.; Tang, S.; Kuo, S. T.; Freitas, D.; Edwards, M.; Wang, H.; Sun, Y.; Yan, X. Lipid Mass Tags via Aziridination for Probing Unsaturated Lipid Isomers and Accurate Relative Quantification. *Angew. Chem.* **2022**, *134* (39), e202207098. DOI: 10.1002/ange.202207098.

5. Hirtzel, E.; Edwards, M.; Freitas, D.; Liu, Z.; Wang, F.; Yan, X. Aziridination-Assisted Mass Spectrometry of Nonpolar Sterol Lipids with Isomeric Resolution. *J. Am. Soc. Mass Spectrom.* **2023**, *34* (9), 1998-2005. DOI: 10.1021/jasms.3c00161.

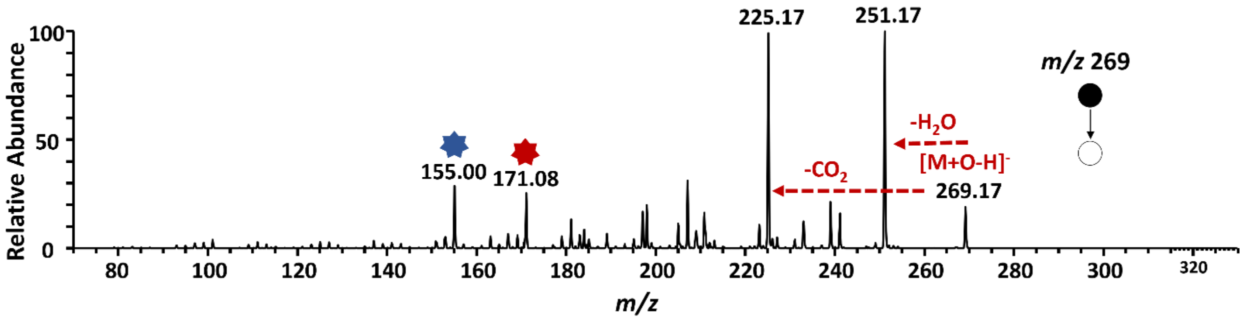

Figure S7. Representative tandem mass spectrum of ions at *m/z* 269 (epoxidation product of palmitoleic acid), showing the double bond diagnostic ions at *m/z* 171 and *m/z* 155.

S7. Changes of C=C bond positional isomer ratios in the GHS-R knockout 5xFAD mouse serum

We have compared the changes of C=C bond positional isomer ratios in the GHS-R knockout 5xFAD mouse serum compared to those in the normal 5xFAD mouse serum (control). Significant differences were found in the C=C bond positional isomer ratios shown in Figure S9.

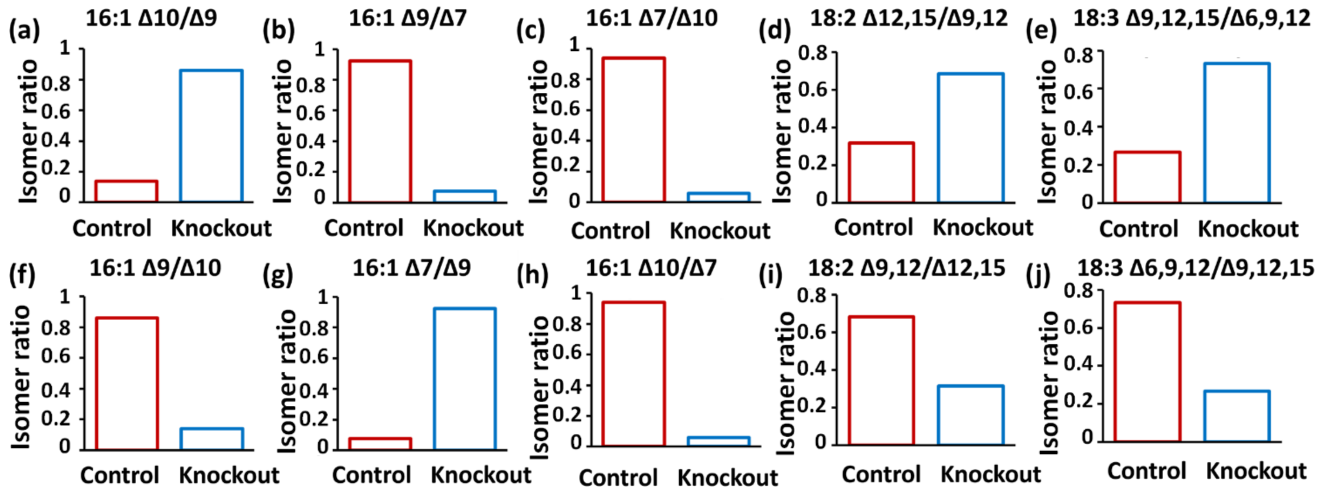

Figure S8. Ratios of C=C bond positional isomers in the GHS-R knockout 5xFAD mouse serum compared to normal 5xFAD mouse serum (control), including (a) FA 16:1(Δ10/Δ9), (b) FA 16:1(Δ9/Δ7), (c) FA 16:1(Δ7/Δ10), (d) FA 18:2(Δ12,15/Δ9,12), (e) FA 18:3(Δ9,12,15/Δ6,9,12), (f) FA 16:1(Δ9/Δ10), (g) FA 16:1(Δ7/Δ9), (h) FA 16:1(Δ10/Δ7), and (i) FA 18:2(Δ9,12/Δ12,15), and (j) 18:3(Δ6,9,12/Δ9,12,15), determined through electro-epoxidation in the theta interfacial microreactor coupled with tandem MS.
